# Measuring Public Occupational Stigma Toward Morticians: Scale Development, Validation, and Implications

**DOI:** 10.3390/bs15121729

**Published:** 2025-12-13

**Authors:** Jiejing Yu, Dongmei Huang

**Affiliations:** School of Philosophy and Sociology, Jilin University, Changchun 130012, China; aliceyujiejing@foxmail.com

**Keywords:** public occupational stigma, mortician, funeral industry, death bias

## Abstract

The present study employed stigma theory to devise a scale measuring public occupational stigma towards morticians and subsequently analyzed its impact. In Phase 1 (*n* = 575), the researchers developed the Public Occupational Stigma toward Morticians Scale (POSMS) and conducted item analysis and exploratory factor analysis. Phase 2 (*n* = 536, *n* = 466) conducted confirmatory factor analysis and retest reliability testing for the scale. Phase 3 (*n* = 1111) validated construct validity, measurement invariance across genders, and criterion-related validity. In summary, these results supported the reliability and validity of the POSMS and confirmed that public occupational stigma toward morticians had negative consequences. This study fills a gap in research on occupational stigma within the funeral industry and offers practical insights for addressing stigma-related issues in other professions.

## 1. Introduction

Occupational stigma has the potential to diminish the appeal of entire industries, causing practitioners to grapple with self-identity issues and encounter interpersonal pressures. Consequently, these sectors continue to experience high attrition rates, which result in talent shortages that constrain industry development (Y. Q. Li et al., 2020). Currently, occupations that scholars are paying particular attention to include call center agents (Shantz & Booth, 2014), food service workers (Shigihara, 2018; Wildes, 2005), veterinarians (Sanders, 2010), nursing home attendants (Jervis, 2001), sanitation workers (Garcia et al., 2017). Despite their significant social value, such professions have a “stigmatized image” in the public eye, which poses a severe threat to practitioners’ sense of identity (Ashforth & Kreiner, 1999; Ashforth et al., 2017). Although research on occupational stigma has made some progress in certain service industries (Y. Q. Li et al., 2020, 2021; Wen et al., 2022), systematic studies on stigma in the funeral industry remain particularly scarce, with most research focusing on the theme of dirty work. Dirty work refers to occupations that are physically, socially, and morally repulsive and tainted (Ashforth & Kreiner, 1999). Physical dirty work refers to occupations where the work environment involves garbage, death, or harmful/hazardous conditions (e.g., morticians). Social dirty work refers to occupations involving frequent contact with stigmatized individuals (e.g., prison guards interacting with inmates) or work characterized by quasi-servile relationships (e.g., social care workers). Moral dirty work encompasses occupations that violate societal moral standards (e.g., tattoo artists) or those where practitioners employ unethical methods such as deception, intrusion, or confrontation (e.g., private investigators) (Ashforth & Kreiner, 1999). Dirty work reflects the objective characteristics of occupations, while occupational stigma is the social stigma imposed on these occupations.

Research on occupational stigma from a public perspective is extremely scarce, whether concerning the funeral industry or other stigmatized groups. Measurements of occupational stigma centered on professional groups include the Occupational Stigma Consciousness Scale adapted by Shantz and Booth (2014) based on Pinel (1999), as well as Occupational Stigma scales developed for nurses (Yang et al., 2022) tour guides (Fan et al., 2024), and physicians (Fan et al., 2022b). Regrettably, most measurements of occupational stigma focus on recipients, with very few targeting implementers. Only the Patient Toward Physician Occupational Stigma Scale (Fan et al., 2022a) adopted an implementer-oriented approach. Guided by the principles of stigma theory (Link & Phelan, 2001), this study addresses the research gaps identified in previous work. Specifically, this study aimed to develop and validate the Public Occupational Stigma toward Morticians Scale (POSMS) to investigate occupational stigma from a public perspective, focusing specifically on funeral service professionals, thereby contributing to research in the field of social stigma.

### 1.1. Stigma Theory

Stigmatization denotes a negative social construct rooted in differences or biases. It represents the collective historical actions through which society has systematically marginalized certain groups. Link and Phelan (2001) seminal study systematically summarized the process and effects of stigma from an intergroup relations perspective. The authors emphasized that the phenomenon involves a combination of five mechanisms: labeling, negative stereotyping, social isolation, status loss, and discrimination (Link & Phelan, 2001). They proposed the following process of stigmatization: First, the mainstream public labels others to highlight differences and links those labeled with negative stereotypes. This leads to the separation of these individuals from the group, resulting in social isolation. Ultimately, this creates unequal status and attitudes. The stigma theory posits that the process of stigmatization begins with the act of labeling and culminates in discrimination and exclusion (Link & Phelan, 2001).

Stigma operates through two distinct pathways, defined by the target and the source: public stigma, which is directed at an individual by others, and self-stigma, which is internalized by the individual (Corrigan, 2004). Public stigma refers to the widespread societal recognition of stigmatization, meaning that large-scale negative perceptions toward a particular group can lead to stereotypes, prejudice, and discrimination (Corrigan, 2004). Many studies on public stigma have examined its impact on self-stigma (Hing & Russell, 2017; Vogel et al., 2013). These studies have indicated that public stigma exerts a profound influence on stigmatized groups. The topic of public stigma has traditionally focused on people with mental disorders (Bazzari & Bazzari, 2023; X. M. Li et al., 2023), problem gambling (Hing & Russell, 2017), substance use (Luo et al., 2024), and Asian Americans (Hwang et al., 2024; Mateer et al., 2024). The stigmatization process has not been thoroughly explored in most studies on occupational stigma (R. R. Zhang et al., 2021).

Occupational stigma, as a key component of stigma research, has historically received significant scholarly attention. The seminal study by E. C. Hughes (1962) established the foundation for research on occupational stigma by introducing the concept of ‘dirty work’. Scholars describe occupational stigma as an “in the eyes of the beholder” phenomenon, which posits that stigma is generated through public observation, reflection, and communication about specific groups, thereby producing distinct negative judgments (J. Hughes et al., 2017). Occupational stigma refers to the attitude or behavior in which individuals holding dominant or majority positions within a specific sociocultural context perceive a particular occupation as dirty and dangerous (physical stigma), shameful and servile (social stigma), or morally corrupt (moral stigma), thereby viewing it as “disgusting” or “loss of face” (Fan, 2023; E. C. Hughes, 1962; J. Hughes et al., 2017). Scholars have found that when society stigmatizes specific occupations, it diminishes the social status of the people who work in them (Hampel & Tracey, 2017; Link & Phelan, 2001; Major & O’Brien, 2005). Accordingly, drawing on the definition of public stigma, we propose the following definition of public occupational stigma: public occupational stigma refers to the stereotypes, prejudices, and discrimination stemming from the public’s negative perceptions of a particular occupation. Therefore, this study examined the dimensions of public occupational stigma toward morticians based on stigma theory (Link & Phelan, 2001).

### 1.2. Public Occupational Stigma Toward Morticians

There has been little quantitative research in China on the occupational stigma associated with morticians, and no studies have yet examined public perceptions of occupational stigma. Morticians are professionals engaged in funeral services who have direct contact with human remains, such as: embalmers, cremation technicians, mortuary cosmetologists, body transporters, and funeral service attendants. Morticians are responsible for a wide range of services, including the preservation of bodies for viewing, the organization of funeral ceremonies and memorial services, and the management of legal documentation. They also play a crucial role in facilitating grief counseling for bereaved families. The funeral profession is responsible for the management of the deceased, yet it has become stigmatized due to the nature of the tasks performed by funeral workers, which often exceed the moral boundaries of societal values (Dick, 2005). Morticians are a marginalized group in society, which renders them vulnerable to discrimination and exclusion. These groups face significant stigmatization.

#### 1.2.1. The Composition of Public Occupational Stigma Toward Morticians in China

In China, public occupational stigma towards morticians has a long history, and this is something that is still very much in evidence today. A survey conducted across five provinces and municipalities in China found that 21.6% of respondents expressed negative attitudes toward morticians (R. Zhang et al., 2017). According to stigma theory (Link & Phelan, 2001), stigma formation is inseparable from five key dimensions. (1) Labeling. The taboo surrounding death in traditional culture intertwines with modern society’s avoidance of life issues, forming the complex psychological foundation of public perception toward the funeral industry (Liang, 2025). (2) Negative stereotypes. Speakers of Chinese often employ the word “yin qi” to describe a sense of ominousness or foreboding. Morticians are associated with an aura of yin qi. The public considers this profession to be jinxed (Jia, 2011). Conflicts over changing funeral practices have led bereaved families to view morticians as people who profit from the dead, resulting in a lack of respect for their profession (Wu, 2022). Professionals in the funeral service industry often face allegations of charging exorbitant fees and engaging in deceptive sales practices, a concern exacerbated by the sector’s uneven quality (He, 2021). (3) Social isolation. Due to secular prejudice and traditional beliefs in China, many morticians lack normal social interactions (Wang, 2023). Morticians face challenges such as high work pressure, interpersonal difficulties, low sense of value recognition, and psychological conflicts regarding marriage and relationships (Zhong, 2021). (4) Status loss. Prior to the Tang Dynasty, the imperial court enacted a strict system of social hierarchy, classifying morticians as members of the lowly class. This status was hereditary, barring them from taking the imperial examinations and prohibiting marriage with commoners. This system did not evolve over time; in subsequent dynasties, morticians continued to occupy a lowly position in society. The low social standing of morticians is primarily due to the constraints of traditional beliefs (Xiong et al., 2006). (5) Discrimination. Stigmatized groups suffer devaluation and discrimination in interpersonal interactions due to the “othering” perpetrated by dominant groups (Hing et al., 2016). Certain influences from traditional Chinese culture have long excluded morticians from mainstream society and subjected them to social discrimination and prejudice (Xiong, 2010).

#### 1.2.2. Public Occupational Stigma Toward Morticians in Other Cultural Contexts

The cultural environment of stigmatized workers shapes the formation of their stigma (Kreiner et al., 2022). As in China, the aforementioned public occupational stigma phenomenon also occurs in other cultures. Death is a social taboo and actively avoided in most cultures (Batista & Codo, 2018; Flynn et al., 2015). South African morticians face varying degrees of social isolation (Mashaba et al., 2025). Society attaches a pervasive occupational stigma to the funeral industry, associating it with death and uncleanliness (Batista & Codo, 2018; Guidetti et al., 2021; Thompson, 1991). Brazilian morticians say the public despises and detests them (Batista & Codo, 2018). Italian morticians feel the stigma manifests in how people perceive their work, as they are often addressed with derogatory terms and avoided in handshakes during greetings (Romao et al., 2025). Sagir (2024) conducted in-depth interviews with Gassals—those who perform ritual bathing of the deceased in Islamic culture. Their work involves physical impurity stemming from direct contact with the corpse, casting a shadow over this profession (Sagir, 2024). Crozier et al. (2025) also conducted in-depth interviews with funeral directors, revealing that individuals outside the industry hold stigmatized and negative stereotypes about funeral directors. The close bond between funeral practitioners and the deceased (Bailey, 2010), along with the practitioners’ economic reliance on death, has led to the stigmatization of funeral work and the development of a sense of stigma associated with it (Lembo, 2022). Some individuals have labeled funeral directors as spreaders of disease, accusing them of profiting from death—a phenomenon that became particularly pronounced during the COVID-19 pandemic (Afifi et al., 2023; Grandi et al., 2024; Mashaba et al., 2025). Funeral work carries a profound social stigma that operates across physiological, social, and moral dimensions (Ashforth & Kreiner, 1999). Consequently, funeral directors are subject to a triple burden of physiological, social, and emotional stress (Jordan et al., 2019). Based on these, the present study focused on the general public to develop a scale measuring public occupational stigma toward morticians and explored its impact, with the aim of eliminating discrimination against the funeral industry.

### 1.3. This Study

This study addressed the research limitations concerning the public occupational stigma toward morticians. Specifically, our goal was to develop and validate a scale that measures the level of mortician stigmatization. In Phase 1, we developed a scale using qualitative materials to assess the public’s occupational stigma toward morticians, employing item analysis and exploratory factor analysis to determine these dimensions. In Phase 2, we further examined the factor structure of the POSMS through confirmatory factor analysis and conducted retest reliability testing. Phase 3 combined samples from Phases 1 and 2 to validate the construct validity, measurement invariance across genders, and criterion-related validity of the scale.

## 2. Phase 1: Development and Evaluation of Scale

### 2.1. Method of Phase 1

#### 2.1.1. Scale Development and Content Validity

A preliminary version of the Public Occupational Stigma toward Morticians Scale was developed by the researchers of this Phase, who based their work on a literature review. The development of this scale entailed the utilization of interview materials from both 16 members of the general public and 14 morticians (See Appendix A), with the incorporation of components from the existing Occupational Stigma Scale. All interview materials were analyzed using thematic content analysis guided by stigma theory (Link & Phelan, 2001). Qualitative research identified five traditional dimensions of public occupational stigma toward morticians: labeling, negative stereotypes, social isolation, status loss, and discrimination. It also revealed a distinct dimension associated with the perceived “dirty work” related to death. Qualitative content analysis provided the foundation for generating an initial item pool. The resulting scale comprised 47 items covering these six dimensions.

Based on the content validity index for scale development (Shi et al., 2012), this Phase invited five experts in occupational stigma research to evaluate the relevance of the questionnaire’s original version on a 1-to-4 scale (1 indicating no relevance, 4 indicating highly relevant). Items with an item-level content validity index (I-CVI) of 0.800 or higher were selected based on the opinions of five experts (See Appendix B). The preliminary version of the Public Occupational Stigma toward Morticians Scale was ultimately developed, comprising five dimensions: negative stereotypes, social isolation, status loss, discrimination, and dirty work, with a total of 27 items. This Phase calculated the average I-CVI score across 27 items, yielding a scale-level content validity index (S-CVI) of 0.881 for the preliminary version of the scale, indicating good content validity (see Table 1).

#### 2.1.2. Participants of Phase 1

The subjects of this Phase were drawn from the general public and were randomly selected via the Credamo data platform. This is China’s professional online survey, behavioral experiment, and data collection platform. This sample was used for project analysis and exploratory factor analysis. A total of 600 questionnaires were distributed for the purpose of this Phase. In order to ensure the integrity of the data, any responses that did not meet the established criteria were excluded from further consideration. The initial phase involved the collection of 575 valid responses, resulting in an overall response rate of 95.833%. It was subsequently designated as Sample 1. The subsequent analysis encompassed two distinct categories of responses: (1) patterned responses and (2) abnormally short or long completion times. The mean age of the participants was 32.550 (*SD* = 7.828); 320 participants (55.700%) were female, and 255 participants (44.300%) were male.

#### 2.1.3. Measures of Phase 1

Participants completed the 27-item Preliminary Version of the Public Occupational Stigma toward Morticians Scale (POSMS). The items encompass five dimensions: negative stereotypes, social isolation, status loss, discrimination, and dirty work. They were rated using a 5-point Likert scale (1 = Strongly Disagree, 5 = Strongly Agree). In this Phase, the Cronbach’s α for the 27-item version was 0.947, while the Cronbach’s α for the 20-item version was 0.935.

### 2.2. Results of Phase 1

#### 2.2.1. Descriptive Statistics and Item Analysis

This Phase employed descriptive statistics and item analysis (see Table 1). First, item analysis was conducted using the item-total correlation method, correlating each item with the total score. All item-total correlation coefficients ranged from 0.302 to 0.762, meeting the criterion of exceeding 0.300. Second, item analysis was conducted using the critical ratio method. Total scores were divided into high and low groups based on the 27% upper and lower thresholds. Independent samples t-tests were performed on the scores of each item for the two groups. Significant differences were found between the high and low groups on all 27 items.

**Table 1:** Descriptive Statistics and Item Analysis for Phase 1 (*n* = 575).

Item	*I-CVI*	*M*	*SD*	*r*	*t*
1	0.800	2.690	1.048	0.673 ***	−20.527
2	0.800	1.410	0.645	0.556 ***	−8.773
3	0.800	1.710	0.727	0.459 ***	−8.275
4	1.000	2.130	1.053	0.691 ***	−16.923
5	1.000	1.930	0.928	0.615 ***	−13.35
6	0.800	1.870	0.887	0.615 ***	−12.915
7	1.000	2.710	1.225	0.597 ***	−21.514
8	0.800	2.220	1.172	0.645 ***	−16.306
9	0.800	2.500	1.298	0.653 ***	−20.761
10	1.000	3.110	1.249	0.611 ***	−21.806
11	0.800	2.070	0.996	0.731 ***	−17.949
12	1.000	1.980	1.058	0.594 ***	−12.783
13	0.800	1.930	1.001	0.755 ***	−16.905
14	0.800	2.080	1.045	0.725 ***	−17.316
15	1.000	2.430	1.268	0.762 ***	−28.403
16	0.800	1.860	0.860	0.747 ***	−16.327
17	0.800	1.830	0.955	0.700 ***	−12.037
18	1.000	1.700	0.884	0.710 ***	−12.241
19	0.800	1.730	0.861	0.697 ***	−12.21
20	1.000	1.960	1.052	0.728 ***	−14.821
21	1.000	2.130	1.140	0.737 ***	−17.389
22	0.800	2.560	1.204	0.721 ***	−23.586
23	1.000	2.030	1.019	0.729 ***	−17.039
24	1.000	2.090	0.996	0.732 ***	−19.465
25	0.800	1.400	0.639	0.532 ***	−7.024
26	0.800	1.450	0.686	0.542 ***	−7.506
27	0.800	1.870	0.920	0.302 ***	−7.379

Note: *** *p* < 0.001.

#### 2.2.2. Exploratory Factor Analysis

This Phase conducted exploratory factor analysis. The results indicated *χ*^2^ = 8647.460, *p* < 0.001, *KMO* = 0.965, demonstrating that the data are suitable for exploratory factor analysis. Then, principal component analysis was applied with a maximum variance rotation to extract four factors with eigenvalues greater than 1, accounting for a cumulative variance contribution of 59.790%. Varimax rotation was selected over oblique rotation (e.g., Promax) to enhance interpretability by producing distinct, minimally correlated factor structures (Sass & Schmitt, 2010). Exclude items based on the following criteria and determine the final number of factors: (1) Delete items 3 and 27 with commonality (extracted common factor variance) below 0.400. (2) Remove items 6, 7, 11, 13, and 16, which exhibit dual loadings exceeding 0.400 with a load difference below 0.150. Perform exploratory factor analysis again on the remaining 20 items. The results showed (see Table 2) that four factors with eigenvalues greater than 1 were extracted, cumulatively explaining 65.793% of the total variance. The factor loadings ranged from 0.592 to 0.789, while the commonality values ranged from 0.537 to 0.741.

Based on the theoretical framework, the four factors were named as follows: Factor 1 comprises 8 items and is named Social Isolation, with an eigenvalue of 4.622 accounting for 23.100% of the variance; Factor 2 comprises 6 items and is named Negative Stereotypes, with an eigenvalue of 3.898 accounting for 19.490% of the variance; Factor 3 comprises 3 items and is named Discrimination, with an eigenvalue of 2.421, accounting for 12.107% of the variance; Factor 4 comprises 3 items and is named Status Loss, with an eigenvalue of 2.217, accounting for 11.086% of the variance.

### 2.3. Conclusion of Phase 1

We analyzed the dimensions of public occupational stigma toward morticians based on stigma theory (Link & Phelan, 2001). This Phase employed item analysis and exploratory factor analysis to develop a preliminary 20-item scale. The Public Occupational Stigma toward Morticians Scale comprises four dimensions: social isolation, negative stereotypes, discrimination, and status loss. The reason this result did not reflect the label dimension is that “funeral services” itself functions as a label, carrying connotations of death and the occupational stigma associated with it. Although the four dimensions of this scale are named after components of the stigma theory, each item incorporates unique stigmas specific to morticians. Next, we would validate the scale to demonstrate its validity.

## 3. Phase 2: Confirmatory Factor Analysis and Retest Reliability

### 3.1. Method of Phase 2

#### 3.1.1. Participants of Phase 2

All subjects in this Phase were members of the general public, with one random sample and one repeat sample collected on the Credamo data platform. A total of 600 questionnaires were distributed. After excluding invalid responses, 536 valid data points were obtained, yielding a response rate of 89.333%, resulting in Sample 2. The mean age of the participants was 32.241 (*SD* = 7.776). The sample comprised 325 females (60.600%) and 211 males (39.400%). After 3 months, a re-test was conducted, yielding 466 recovered data points for the sample, resulting in Sample 3. The mean age of the participants was 32.180 (*SD* = 7.544); 289 participants were female (62.000%), and 177 were male (38.000%).

#### 3.1.2. Measures of Phase 2

Participants completed the initial version of the 20-item public occupational stigma toward morticians scale (POSMS). The scale comprises four dimensions: negative stereotypes, social isolation, status loss, and discrimination. Responses were rated on a 5-point Likert scale (1 = Strongly disagree, 5 = Strongly agree). 

### 3.2. Results of Phase 2

#### 3.2.1. Confirmatory Factor Analysis

This Phase conducted confirmatory factor analysis (see Table 3). To examine whether the four-factor model represents the optimal theoretical framework, separate models were constructed: a single-factor model, a two-factor model (combining social isolation and status loss into one dimension, and negative stereotypes and discrimination into another), a three-factor model (treating social isolation and negative stereotypes as separate dimensions, while merging discrimination and status loss into one dimension), and a four-factor model. Results indicated that the fit indices for the single-factor, two-factor, and three-factor models failed to meet psychometric requirements. The four-factor model demonstrated good fit, with item loadings ranging from 0.590 to 0.855 (*p* < 0.010). Therefore, the four-factor model represents the optimal fit.

#### 3.2.2. Reliability Analysis

This Phase conducted reliability analysis and administered retests after four weeks. Results indicate (see Table 4) that reliability coefficients ranged from 0.781 to 0.953, split-half reliability coefficients ranged from 0.749 to 0.913, and test–retest reliability coefficients ranged from 0.773 to 0.930. All these results exceeded the standard of 0.700.

### 3.3. Conclusion of Phase 2

This Phase employed confirmatory factor analysis and reliability analysis on the 20-item Public Occupational Stigma Towards Morticians scale. The results of the confirmatory factor analysis indicated that a four-factor model provided the most adequate fit for the scale. Reliability analysis further demonstrated adequate internal consistency for the total scale and its four subscales: social isolation, negative stereotypes, discrimination, and status loss.

## 4. Phase 3: Questionnaire Validity Testing

### 4.1. Method of Phase 3

#### 4.1.1. Participants of Phase 3

This Phase combined the two random samples (Sample 1 and Sample 2) from Phase 1 and Phase 2 to form Sample 4, comprising a total of 1111 valid data points. The mean age of the participants was 32.400 (SD = 7.801); 644 participants (58.000%) were female, and 467 participants (42.000%) were male.

#### 4.1.2. Measures of Phase 3

Public occupational stigma toward morticians scale This 20-item scale comprises four dimensions: negative stereotypes, social isolation, status loss, and discrimination. It employs a 5-point Likert scale (1 = Strongly Disagree, 5 = Strongly Agree). Higher individual scores indicate more severe occupational stigma toward morticians. In Sample 4, this scale achieved a Cronbach’s α of 0.939.

Perceived devaluation discrimination scale This scale was developed by Link et al. (1987) to measure public devaluation of individuals with mental illness. For this Phase, eight items were selected and adapted by replacing “mental illness” with ‘mortician’ to measure public devaluation of morticians. The scale uses a 5-point Likert scale, with responses from 1 to 5 indicating “Strongly Disagree” to “Strongly Agree.” In Sample 4, this scale yielded a Cronbach’s α coefficient of 0.875.

Social Distance Scale This Phase employed the 12-item Social Distance Scale (Norman et al., 2008), which uses a 5-point Likert scale ranging from “very unwilling” to “very willing” (scores 1–5). Higher scores indicate closer social distance. In Sample 4, the Cronbach’s α for this scale was 0.950.

### 4.2. Results of Phase 3

#### 4.2.1. Construct Validity

This Phase employed Sample 4 for construct validity analysis (see Table 5). The convergent validity analysis results indicated that the average variance extracted (AVE) for the four dimensions ranged from 0.527 to 0.606, exceeding 0.400; composite reliability (CR) ranged from 0.779 to 0.925, exceeding 0.700. Therefore, this scale demonstrated good convergent validity. The discriminant validity analysis demonstrated that the heterotrait/monotrait ratio of correlations (HTMT) ranged from 0.499 to 0.782, which is below the 0.850 threshold. Thus, the scale demonstrated adequate discriminant validity.

#### 4.2.2. Testing for Measurement Invariance Across Genders

This Phase employed Sample 4 to conduct Testing for Measurement Invariance Across Genders (see Table 6). The results indicated that in the Configural Invariance Model (M1), Metric Invariance Model (M2), Scalar Invariance Model (M3), and Strict Invariance Model (M4), all fit indices meet psychometric requirements, satisfying the prerequisites for conducting equivalence testing. In the comparisons between M2 and M1, M3 and M2, and M4 and M3, ∆*CFI* < 0.010 and ∆*RMSEA* < 0.015, the results met the criteria for measurement invariance (Cheung & Rensvold, 2002). Therefore, this scale had Measurement Invariance Across Genders.

#### 4.2.3. Criterion-Related Validity

This Phase employed Sample 4 for correlation analysis (see Table 7). Results indicated that the total score of public occupational stigma and its respective dimensions showed a significant negative correlation with social distance (*p* < 0.001) and a significant positive correlation with devaluation (*p* < 0.001).

### 4.3. Conclusions of Phase 3

This Phase further validated the construct validity and cross-gender consistency of the 20-item public occupational stigma toward morticians scale, confirming the validity of its four dimensions. In addition, this Phase examined public devaluation and social distance toward morticians. Correlation analysis indicated that public occupational stigma toward morticians, devaluation and social distance were significantly correlated, thereby demonstrating that the POSMS possesses good external validity.

## 5. General Discussion

As public occupational stigma toward morticians intensifies, it has the potential to impact the relationship between the two parties and even influence individuals’ career choices in the field. In this study, we developed and validated the POSMS: a 20-item Public Occupational Stigma Scale designed to assess public occupational stigma toward morticians or workers in the death industry. In Phase 1 and Phase 2, we explored and validated a measurement model of public occupational stigma toward morticians. Our results indicated that the internal consistency of the scale and its subscales reached a level ranging from good to excellent, while also confirming the scale’s construct validity and predictive validity. In Phase 3, we confirmed that the concept-related variables (devaluation and social distance) showed significant correlations. In summary, we encourage future researchers to investigate variables and phenomena directly or indirectly related to POSM in order to improve social impressions of minority groups.

### 5.1. The Measurement Model of Public Occupational Stigma Toward Morticians

We examined the measurement structure of public occupational stigma toward morticians by testing several different models. Specifically, we tested single-factor, two-factor, three-factor, and four-factor models, finding that the four-factor model provided the best fit. In addition, we confirmed that the POSMS possesses good construct validity and test–retest reliability.

The dimensions in this study were derived from stigma theory (Link & Phelan, 2001): social isolation, negative stereotyping, discrimination, and status loss. (1) Social isolation. Social isolation is a very classic dimension. For example, I wouldn’t shake hands with a mortician or dine with one. This fully illustrates why we use the term “isolation” rather than “distance.” Many members of the public we interviewed believe that funeral directors carry an odor, or that their hands retain a mysterious aura from handling the deceased—even after thoroughly washing them. Some argue that having a family member who is a mortician could create strain, and that the occupational stigma also affects the family members themselves. (2) Negative stereotyping. Research has established that stereotypes play a key role in stigmatization, thereby fueling discrimination (Granjon et al., 2024). The funeral industry is deeply rooted in specific cultures, and different cultures impose distinct expectations and constraints upon funeral practitioners (Verhoeven et al., 2025). In China, funeral rites remain a taboo subject that most people are reluctant to broach (He, 2021). Many people believe that morticians work in a profession permeated by yin qi, and they fear that this energy may adhere to them through contact. (3) Discrimination and status loss. Throughout most of China, Taoist priests who perform funeral rites command deep respect. Conversely, morticians who handle the bodies frequently face contempt and disgust from a society that views them as inferior (He, 2021). Some members of the public hold the perception that morticians engage in work that is both undignified and unclean (Jia, 2011).

The fundamental professional aspect of the funeral industry entails confronting death and suffering (Guidetti et al., 2025). Based on stigma theory, we found that public occupational stigma toward morticians followed this framework, yet each dimension revealed the commonality of “death.” According to the Stigma Theory proposed by Link and Phelan (2001), transformative strategies must ultimately address the root causes of stigmatization. The theory asserts that these strategies must alter the entrenched perceptions and beliefs of dominant groups, which are precisely the attitudes that lead to labeling, stereotyping, exclusion, devaluation, and discriminatory practices. Drawing an analogy from research on sex workers (Benoit et al., 2020), the stigma attached to death and corpses may be an unalterable constant. The very nature of the funeral director’s profession is associated with “death,” casting a veil of “stigma” over it. Researchers have made similar findings among abortion providers (O’Donnell et al., 2011). As long as people continue to shun death, they will look down on morticians (Jia, 2011). However, as society continues to develop and progress in China, people’s perspectives are gradually evolving, and attitudes toward funerals are also changing with the times, with public stigma diminishing (He, 2021). Therefore, incorporating the POSMS scale and its subscales into research not only facilitates a deeper understanding of the public occupational stigma toward morticians but also provides a more comprehensive quantitative perspective for studies dedicated to mitigating the effects of occupational stigma.

### 5.2. Limitations and Future Directions

The POSMS represented the inaugural endeavor to quantify the public occupational stigma associated with morticians, and the results demonstrated the scale’s potential utility in the field of stigma research. We hope POSMS will advance quantitative research in this field and uncover the causes and mechanisms influencing the public occupational stigma toward morticians. We note that this study is a preliminary exploration of occupational stigma in the funeral industry; accordingly, we outline several of its limitations below. Firstly, a primary limitation is that all data in this study were self-reported, a method inherently subject to potential biases. Intergroup contact has been demonstrated to be an effective method of reducing public stigma. Future research should employ experimental methods to examine and mitigate occupational stigma toward funeral directors. Secondly, the public sample in this study originated from China and exhibits cultural limitations. However, as indicated in the literature review, the funeral industry faces varying degrees of stigma globally. Future research could adapt the project design to accommodate more diverse cultural contexts. This study did not present the stigma of “death” as a separate dimension. This may be due to the fact that the core of all four dimensions revolves around “death”. We recommend that future research consider the possibility of “death” exerting a more independent influence and modify the scale as needed for subsequent studies. Concurrently, future research endeavors may also contribute to the dissolution of the taboo by conducting immersive ethnographic experiences.

Despite the limitations of the study, we believe our research represents a bold step forward in the study of occupational stigma in the public sphere. Research confirms that silence amplifies societal biases (Szekeres et al., 2023). Despite the fact that this industry provides essential services, it has long been the subject of negligence (Guidetti et al., 2025). This study has two distinguishing features. Firstly, it constitutes a quantitative investigation into public occupational stigma. Secondly, it is a specific investigation into public occupational stigma targeting particular professions. POSMS helps quantify societal attitudes toward morticians, which is crucial for understanding occupational stigma among the public. Future research may utilize the POSMS scale to validate the effectiveness of stigma interventions, thereby effectively alleviating occupational stigma. Indeed, the function of stigma is more akin to that of a colored label affixed to stigmatized groups. As society continues to operate, these labels may either proliferate or diminish, influenced by a multitude of factors. Nevertheless, the prevalence of these labels is likely to diminish or even vanish entirely. Therefore, this research focuses on understanding what labels are and how deeply ingrained they are. Despite the fact that POSMS is currently limited in scope, in that it is merely a measurement scale, it provides a quantitative tool for reducing color-coded labels. We hope these findings contribute to reducing occupational stigma, however modestly.

### 5.3. Conclusions

Although this represents only the first step in quantitative research within the funeral industry, the present study confirmed the reliability and validity of the Public Occupational Stigma toward Morticians Scale. The subsequent identification of four dimensions of public occupational stigma towards morticians provides a quantitative foundation for the development of interventions to reduce occupational stigma in this profession. This approach facilitates the observation of the evolution of public occupational stigma, thereby contributing to the enhancement of interpersonal relationships between the public and funeral directors.

## Figures and Tables

**Table 2:** Exploratory Factor Analysis Factor Loadings and Commonality (*n* = 575).

	Item	Factor 1	Factor 2	Factor 3	Factor 4	Commonality
1	The working environment of a mortician makes me feel uncomfortable.	0.352	**0.620**	0.135	0.100	0.537
2	Morticians are considered inferior people.	0.099	0.230	**0.697**	0.311	0.646
3	Morticians have a bad reputation.	0.317	0.340	0.161	**0.613**	0.617
4	Morticians have low social status.	0.140	0.208	0.311	**0.754**	0.727
5	Morticians make money off the dead.	0.174	**0.646**	0.253	0.166	0.539
6	A mortician needs to be resilient/have a strong destiny	0.207	**0.785**	0.09	0.08	0.673
7	Morticians have many taboos.	0.169	**0.794**	−0.084	0.182	0.699
8	The profession of mortician is not respected.	0.238	0.148	0.146	**0.789**	0.723
9	The profession of mortician frequently involves exposure to dirty things.	0.258	**0.592**	0.305	0.299	0.599
10	The profession of mortician carries a heavy yin qi.	0.311	**0.773**	0.140	0.168	0.741
11	If my family were morticians, it would be a burden for me.	**0.645**	0.192	0.335	0.204	0.607
12	I won’t have dinner with a mortician.	**0.682**	0.192	0.401	0.123	0.678
13	I won’t be friends with a mortician.	**0.726**	0.143	0.343	0.151	0.688
14	I would never invite a mortician to a wedding reception.	**0.752**	0.254	0.256	0.097	0.705
15	I would never invite a mortician to a newborn’s 100-day celebration.	**0.772**	0.28	0.143	0.137	0.713
16	I won’t date a mortician.	**0.609**	0.443	−0.064	0.311	0.667
17	I don’t initiate handshakes with morticians.	**0.663**	0.322	0.209	0.191	0.623
18	I will maintain social distance from a mortician.	**0.672**	0.350	0.034	0.290	0.659
19	I look down on morticians.	0.299	0.055	**0.748**	0.159	0.677
20	I despise morticians.	0.356	0.082	**0.704**	0.098	0.638

Note: Bold text indicates that the item meets the exploratory factor analysis criteria and belongs to the factor in that column.

**Table 3:** Comparison of Model Fit in Confirmatory Factor Analysis (*n* = 536).

Model	*χ* ^2^	*df*	*χ*^2^/*df*	*RMSEA*	*CFI*	*TLI*	*SRMR*
Single-factor model	1268.555	170	7.462	0.110	0.810	0.788	0.071
Two-factor model	1172.787	169	6.940	0.105	0.826	0.805	0.074
Three-Factor Model	755.075	167	4.521	0.081	0.898	0. 884	0.060
Four-Factor Model	446.295	163	2.738	0.057	0.951	0.943	0.042

**Table 4:** Reliability Coefficients of the Public Occupational Stigma Scale.

	Total	Social Isolation	Negative Stereotypes	Discrimination	Status Loss
Cronbach’s α (*n* = 536)	0.943	0.930	0.864	0.830	0.781
Split-Half (*n* = 536)	0.880	0.913	0.844	0.820	0.749
Test–Retest (*n* = 466)	0.930	0.913	0.849	0.776	0.773

**Table 5:** Construct Validity of the Public Occupational Stigma Scale (*n* = 1111).

	AVE	CR	1.1	1.2	1.3	1.4
1.1 Social isolation	0.606	0.925	1			
1.2 negative stereotypes	0.527	0.869	0.782	1		
1.3 Discrimination	0.561	0.793	0.711	0.499	1	
1.4 status loss	0.540	0.779	0.741	0.710	0.691	1

**Table 6:** Results of Measurement Invariance Across Genders (*n* = 1111).

	*χ* ^2^	*df*	*χ*^2^/*df*	*RMSEA*	*CFI*	*TLI*	*SRMR*	∆*RMSEA*	∆*CFI*
M1	573.324	276	2.077	0.044	0.977	0.969	0.039		
M2	594.747	292	2.037	0.043	0.977	0.970	0.042	0.001	0.000
M3	622.954	308	2.023	0.043	0.976	0.970	0.042	0.000	0.001
M4	662.693	328	2.020	0.043	0.974	0.970	0.044	0.000	0.002

Note: M1 = Configural Invariance Model, M2 = Metric Invariance Model, M3 = Scalar Invariance Model, M4 = Strict Invariance Model.

**Table 7:** Correlation Analysis for Phase 3 (*n* = 1111).

	*M*	*SD*	1	1.1	1.2	1.3	1.4	2
1 public occupational stigma	41.426	14.174	1					
1.1 Social isolation	16.135	6.650	0.931 ***	1				
1.2 negative stereotypes	15.018	5.518	0.875 ***	0.701 ***	1			
1.3 Discrimination	4.244	1.763	0.671 ***	0.606 ***	0.421 ***	1		
1.4 status loss	6.029	2.563	0.769 ***	0.628 ***	0.580 ***	0.543 ***	1	
2 devaluation	20.154	6.160	0.667 ***	0.599 ***	0.584 ***	0.375 ***	0.616 ***	1
3 Social distance	44.930	9.430	−0.800 ***	−0.821 ***	−0.670 ***	−0.454 ***	−0.541 ***	−0.655 ***

Note: *** *p* < 0.001.

## Data Availability

The datasets generated and/or analyzed during the current study are not publicly available due to the university’s policy but are available from the corresponding author on reasonable request.

## Appendix A. Basic Information of In-Depth Interview Participants

**Table A1:** Basic Information for the Public (*n* = 16).

	Gender	Age	Occupation	Note
F01	Male	25	Freelance	Relatives working in the funeral industry
F02	Female	30	Accountant	
F03	Female	37	Nurse	
F04	Female	52	Resident Advisor	
F05	Male	24	Delivery Driver	Relative working in the funeral industry
F06	Male	67	Security Guard	
F07	Female	41	Pharmacy Clerk	
F08	Male	50	Farmer	
F09	Male	50	Middle School Teacher	
F10	Male	59	Retired	
F11	Male	52	Government Agency	
F12	Female	56	Retired	Friend working in the funeral industry.
F13	Female	53	Retired	
F14	Female	68	Retired	
F15	Female	42	Research Institute	
F16	Female	28	Police Officer	Relative working in the funeral industry

**Table A2:** Basic Information for Morticians (*n* = 14).

	Gender	Age	Category	Years of Service	Income (RMB)
S01	Female	25	Mortuary Restoration	3	5000–7000
S02	Male	30	Mortuary Cosmetology	10	5000–6000
S03	Male	29	Embalming	10	5000–6000
S05	Female	27	Encoffinment	2	20,000–30,000
S06	Female	23	Encoffinment	0.3	2000
S07	Male	26	Funeral Escort	26	6000
S08	Male	30	Encoffinment	0.5	confidential
S09	Female	26	Mortuary Restoration	3	4000
S10	Male	35	Encoffinment	16	10,000
S11	Male	24	Encoffinment	3	5000–10,000
S12	Male	28	Mortuary Restoration	2	7000–8000
S13	Female	27	Embalming	2	confidential
S15	Female	30	Encoffinment	11	6000–8000
S16	Female	23	Mortuary Cosmetology	3.5	6000–7000

Note: In Chinese, the number 4 is homophonous with the word for “death,” so morticians’ numbers never include the digit 4.

## Appendix B. List of Content Validity Experts

**Table A3:** List of Content Validity Experts (*n* = 5).

	Degree	Professional	Research Areas	Organization	Title
J01	Doctor	Health Services Management	Occupational stigma	Northeastern University (China)	Professor
J02	Doctor	Human Resource Management	Occupational stigma	Jiangsu University	Professor
J03	Doctor	Tourism Management	Occupational stigma	Southwestern University of Finance and Economics	Professor
J04	Doctor	Tourism Management	Occupational stigma	Jishou University	Lecturer
J05	Doctor	Business Administration	Occupational stigma	Xihua University	Associate Professor
